# Covid-19 Vaccination and the issue of insurance status

**DOI:** 10.1093/eurpub/ckac129.548

**Published:** 2022-10-25

**Authors:** D Schröpfer, R Fortunato, N Devaud, R Koppensteiner

**Affiliations:** SAD, Stadt Zürich Stadtärztlicher Dienst, Zürich, Switzerland

## Abstract

**Issue:**

It is estimated, that 11000 - 14000 people that live in Zurich do not have health insurance. For the vaccination against Covid-19 in Switzerland one had to register oneself on a website (Vacme) with your address and a Swiss health insurance number was required. This systematically excluded any people without insurance or Sans Papiers.

**Description:**

To address this issue the city physician service Zurich organized a vaccination program for the uninsured from 14.06.2021 onward. To achieve this a code which could be used instead of the regular insurance number was organized and the staff recorded personal information of patients on site to remove language and technological barriers. Furthermore, the Police department was informed and agreed not to circulate the area of the vaccination service on vaccination days to minimize access-barriers for patients without legal residency.

**Results:**

Between 14.06.2021 and 19.02.2022, 880 people came for vaccination of which 603 were clearly identifiable as individuals from vulnerable populations with no insurance. After initial organization, other vaccination centers in Switzerland were able to use the substitute number to vaccinate people without health insurance as well..

**Lessons:**

There is a sizeable amount of people living without registration or/and health insurance. It is therefore critical not to forget these people when organizing public health measures, especially when addressing a pandemic or other infectious diseases (HIV, Hep C). Non-insurance is a known issue for universal access to care.

Acknowledgements: We would like to thank the hold crew of the ambulatorium kanonengasse at SAD

**Key messages:**

• When fighting a pandemic it is of utmost importance to reach as many people as possible.

• Acess must be possible also for vulnernable people.

